# Adipogenic Differentiation of Muscle Derived Cells is Repressed by Inhibition of GSK-3 Activity

**DOI:** 10.3389/fvets.2018.00110

**Published:** 2018-06-12

**Authors:** Zoe Redshaw, Paul Thomas Loughna

**Affiliations:** ^1^School of Veterinary Medicine and Science, The University of Nottingham, Loughborough, United Kingdom; ^2^Faculty of Health and Life Sciences, De Montfort University, Leicester, United Kingdom

**Keywords:** Wnt, skeletal muscle, GSK-3β, PPARγ, adipogenesis

## Abstract

Intramuscular fat is important in large animal livestock species in regard to meat quality and in humans is of clinical significance in particular in relation to insulin resistance. The canonical Wnt signalling pathway has been implicated at a whole body level in regulating relative levels of adiposity versus lean body mass. Previously we have shown that pig muscle cells can undergo adipogenic differentiation to a degree that is dependent upon the specific muscle source. In this work we examine the role of the canonical Wnt pathway which acts through inactivation of glycogen synthase kinase-3 (GSK-3) in the regulation of adipogenic differentiation in muscle cells derived from the pig semimembranosus muscle.

The application of lithium chloride to muscle derived cells significantly increased the phosphorylation of GSK-3β and thus inhibited its activity thus mimicking Wnt signaling. This was associated with a significant decrease in the expression of the adipogenic transcription factor PPARγ and an almost complete inhibition of adipogenesis in the cells. The data also suggest that GSK-3α plays, at most, a small role in this process.

Studies *in vivo* have suggested that the Wnt pathway is a major regulator of whole body adiposity. In this study we have shown that the ability of cells derived from porcine skeletal muscle to differentiate along an adipogenic lineage, *in vitro,* is severely impaired by mimicking the action of this pathway. This was done by inactivation of GSK-3β by the use of Lithium Chloride.

## Introduction

Skeletal muscle is the largest organ of the body comprising >45% of overall mass and is the major contributor to whole-body protein metabolism. This tissue also demonstrates remarkable plasticity and may be subject to rapid changes in mass. The size of multinucleate muscle fibers is dictated by a number of factors the predominant being nutrition and mechanical loading. Muscle fibers also have a remarkable ability to regenerate following trauma or disease. Both muscle hypertrophic growth and regenerative capacity are, at least in part, dependent upon resident stem cell populations.

Intermuscular adipose tissue (IMAT) has been described to include fat that is located beneath the muscle fascia and within the muscle itself (1). This intramuscular fat, deposited between skeletal muscle fibres and bundles of muscle fibers is highly evident in large animals and humans although insignificant in healthy mice and rats. In livestock the degree of intramuscular adipose tissue (marbling) varies between species and breeds within a species and it is a major contributor to meat quality. In humans there is increased accretion of adipose tissue within skeletal muscle with age, inactivity and disease conditions such as diabetes (1, 2) . The increase in intramuscular adipose tissue in humans is visible on MRI images and has a clinical impact with regard to insulin resistance and cardio-metabolic syndrome. There are number of different cell types present in skeletal muscle that could contribute to fat cell formation including satellite cells, mesenchymal progenitors, fibro/adipogenic progenitors (FAPs) and pericytes (1, 3, 4). *In vitro* studies have shown that satellite cells isolated from skeletal muscle or resident beneath the basil lamina of isolated muscle fibers can undergo adipogenic differentiation when subjected to high glucose levels or thiazolidinediones which are potent activators of the adipogenic marker PPARγ (5, 6). We have recently shown that cellsderived from different porcine skeletal muscles differ distinctly in their adipogenic potential. Cells derived from the diaphragm had a dramatically reduced ability to undergo adipogenic differentiation when compared to those derived from the hindlimb semi-membranosus muscle (7, 8). The Wnt family of secreted protein growth factors play a significant role in the differentiation and growth of skeletal muscle cells (9). Activation of the canonical Wnt pathway through the administration of lithium chloride has been shown to induce myotube hypertrophy (10). In other cell types Wnt signalling has been shown to have a significant role in differentiation with reduced signalling causing a shift in cell fate of pre-osteoblasts from osteoblasts to adipocytes (11). The muscles of Wnt 10b ^-/-^ mice show impaired regenerative capacity following injury with replacement of muscle by adipose tissue (12). Satellite cells isolated from obese Zucker rats expressed an increased propensity to adipogenesis (as measured by oil red O) when compared to those from lean rats and this correlated with reduced Wnt 10b expression (13). These studies although suggestive do not however directly demonstrate a role for canonical Wnt signalling in the trans-differentiation of muscle stem cells along an adipogenic lineage. In the present study we have employed a large animal (porcine) model to study the effects of activation of the Wnt canonical signalling pathway upon adipogenesis in muscle derived cells.

## Materials and Methods

Porcine muscle derived stem cells were isolated from the diaphragm (DIA) and hind limb semi-membranosus (SM) muscles, as previously described (7, 8). Freshly isolated cells were cultured on type I collagen (0.01%, Sigma, UK) coated plastic, in Memα growth media [GM, 20% FBS (Invitrogen, UK), 2 mM L-glutamine (Sigma), 100 IU/ml penicillin, 100 µg/ml streptomycin, 3 µg/ml amphotericin B (Invitrogen)] and incubated at 37°C in a humidified atmosphere in 5% CO2, 90% Nitrogen and 20% O2 until approximately 60% confluent before cryogenic preservation in FBS and 10% DMSO (invitrogen).

### Myogenic and Adipogenic Differentiation

Cells were seeded at a density of 2.6 × 104 cells/cm2 on collagen coated plastic and initially cultured in GM until ~80% confluent (incubated at 37°C, 5% CO2 and air). At this stage, GM was substituted for either myogenic (Myo, DMEM, 2% horse serum, 2 mM L-glutamine, 100 IU/ml penicillin, 100 µg/ml streptomycin, 3 µg/ml amphotericin B) or adipogenic differentiation media (Ad, DMEM, 10% horse serum (Invitrogen), 1 µM dexamethasone (DEX, Sigma), 50 µM IBMX (Sigma), 10 µU insulin (Eli Lilly & Co Ltd, UK) and L-glutamine, penicillin, streptomycin and amphotericin B as for Myo). For RT-PCR, cells were cultured up to +24 h differentiation prior to RNA isolation, or for LiCl studies up to day 6 of differentiation, with Ad media added as described for 3 days, followed by 3 days in Ad media minus DEX and IBMX). For drug treated cells, 20 mM LiCl (Sigma) was added to Ad media on days 0 and 3 only. Following differentiation at day 6, cells were initially fixed in 70% ethanol.

### Comparison of Serum Type on Adipogenic Differentiation

Adipogenic differentiation was induced [as previously described (7, 8)] using Ad media containing either 10% foetal bovine serum (FBS, Invitrogen) or 10% horse serum. All other conditions remained identical for both treatments.

### Oil Red O Staining

Prior to Oil Red O staining, cells were additionally fixed in 10% buffered formalin for 20 min and lipid was visualised via Oil Red O (0.5%, Sigma) staining as described previously (7, 8).

### Image Analysis

All images used for the quantification of Oil Red O staining, were analysed using Image Pro version 6.3. Phase contrast images were prepared as tiff documents for 100× magnification, taken for all experiments carried out at *n* = 6, with 15 fields of view (FOV) per replicate. For individual staining analyses, a macro was created to include the range of colour intensities denoting a positive result and applied to all grouped images. Error bars represent SD.

### Semi-Quantitative RT-PCR

Total RNA was isolated using the Qiagen mini prep kit (Qiagen, UK) and reverse transcription performed using superscript™ first strand synthesis system (Invitrogen) according to the manufacturers guidelines (input RNA: 500 ng). Samples were treated with DNA free dnase kit (Applied Biosystems, UK) and sample purification was ascertained via nanodrop, with all samples having a 260/280 OD ratio greater than 2.0. All primers were designed against known pig mRNA sequences (Ensembl Genome Browser) using free software Primer3 (http://frodo.wi.mit.edu/). Primer sequences: Wnt10b (111 bp) forward: AATGCGAATCCACAACAACA, reverse: CTCCAGCACGTCTTGAACTG. Amplification conditions as described in (8).

### SDS-PAGE/western Blotting

Cell lysates were prepared and electrophoresed as described in (7, 14). Protein was generated at four time points: undifferentiated (70% confluence), Day 1, Day 3 and Day 6 following induction of differentiation, in triplicate for each treatment. Primary antibodies were rabbit monoclonal antibodies to PPARγ (1:300, Abcam), GSK3β (1:1,000, Cell Signalling, UK), pGSK3β (1:1,000, Cell Signalling), pGSK3α (1:1,000, Cell Signalling) and mouse monoclonal antibody α-Tubulin (1:1,000, Abcam). Secondary antibodies were anti-rabbit HRP and anti-mouse HRP (1:2,000, Cell Signalling). Quantification of protein expression was analysed via densitometry using ImageQuant TL (version 2005, Amersham Biosciences).

### Statistical Analysis

Where applicable, student’s *t*-tests were performed on data sets (unpaired, two-tailed). Error bars represent SD.

## Results and Discussion

Muscle stem cells including the predominant satellite cell population have been shown to have the capability to trans-differentiate along an adipogenic lineage including on isolated muscle fibers. In contrast a study in mice by Joe et al (4). strongly suggested that FAPs are the main source of adipocytes in adult tissue and another study, also in mice, showed a major role for mesenchymal progenitors in fat cell formation (3) A recent lineage study on myofibers isolated from mouse masseter and hind-limd muscles suggested that non-myogenic fates are a result of myofiber-associated progenitors rather than mesenchymal stem cells (15). Further study will be needed to define the cell types involved in the pig cells.We have recently shown that cells derived from different muscles have a distinctly different capacities to undergo adipogenic differentiation; with those isolated from the hind-limb semi-membranosus (SM) muscle being able to undergo significantly greater adipogenic differentiation when compared to those isolated from the diaphragm (DIA) (7, 8). This is in keeping with previous observations that different skeletal muscles respond differently to the same stimulus (16, 17).

**Figure 1:**
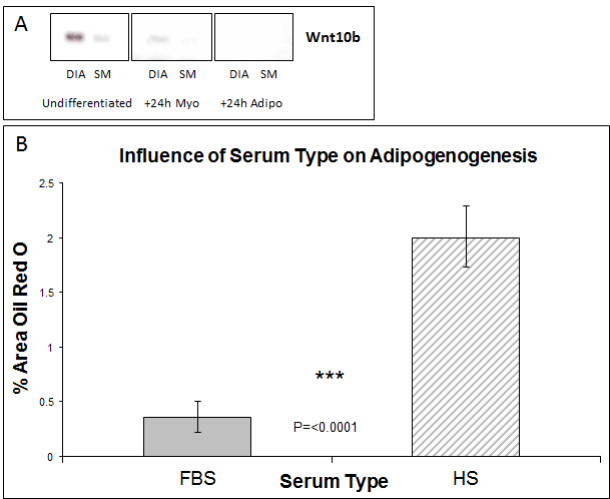
Wnt Expression and The influence of serum type on adipogenesis. **(A)** Expression of Wnt 10b by RT-PCR in muscle stem cells derived from diaphragm (DIA) and semi-membranosus (SM) muscles: undifferentiated and +24 h myogenic and adipogenic differentiation. **(B)** Lipid accumulation was measured by Oil Red O staining at Day 6 of differentiation, for cells grown in media containing either foetal bovine serum (FBS) or horse serum (HS). (*n* = 6, 15 FOV/rep). Error bars represent SD.

The Wnt (wingless type mouse mammary tumour virus integration-site family) family of growth factors play a crucial role during development in cell specification. The Wnt genes encode a large family of proteins that can signal through both canonical and non-canonical pathways (18). Of particular interest is Wnt 10b acting through the canonical pathway. This protein binds to the Frizzled/LRP receptor complex which leads to the inactivation of GSK3-β and the accumulation of cytosolic β-catenin which then translocates to the nucleus to activate transcription of Wnt responsive genes which include the myogenic regulatory factors MyoD and myogenin (19). In Wnt 10b^−/−^ mice regeneration of muscle after induced muscle injury is associated with accumulation of large amounts of lipid and myoblasts from these animals express high levels of adipogenic markers such as PPAR-γ (12). Furthermore Wnt 10b mRNA levels are reduced in myoblasts from 24 month old compared to 8-month-old mice with cells from the older mice also exhibiting a greater adipogenic potential (12). It is not known, however, whether this factor plays any role in the markedly different adipogenic potential of stem cells derived from different muscles that we have described previously (7, 8). We therefore examined the expression of the Wnt 10b transcript in cells derived from the DIA and SM. Cells derived from the SM (which have a greater adipogenic potential) have a lower level of the 10b transcript just prior exposure to DM (i.e., at 80% confluence) compared to those from the DIA (Figure 1A). In cells exposed to myogenic DM for 24 h this higher expression in the DIA derived cells was maintained. In cells exposed to adipogenic DM, however, the transcript was not detectable in cells derived from either muscle (Figure 1A). These data suggest that the adipogenic potential of a muscle cell population(s) might be inversely correlated to levels of endogenous activation of the canonical Wnt pathway. If this is the case then activation of the canonical Wnt pathway should suppress the adipogenic transdifferentiation of these cells.

Subsequent studies were carried out on cells derived from the SM only as we have shown previously that cells derived from the diaphragm have a very limited potential for adipogenic differentiation. Cells were isolated from the SM muscle and the predominant satellite cell population identitied verified by the expression of myogenic regulatory factors Pax7 and Pax3 (8). The majority of cells expressed both markers, which we typically find to be greater than a 98% pure population [(7, 8) and data not shown]. Having previously shown that cells from SM muscle are capable of adipogenic differentiation (7, 8), we wished to further enhance this response. We therefore carried out cell culture studies at 20% oxygen concentration rather than at lower (~5%) more physiological levels which although enhancing myogenic retards adipogenic differentiation (7). We also compared the effect of serum type, within the adipogenic DM, on the ability of cells to generate lipid. Many adipogenic-inducing protocols use media containing 10% foetal bovine serum (FBS) as standard, compared with myogenic differentiation where horse serum (HS) is preferentially used to induce terminal differentiation. Following adipogenic differentiation, we found that choice of serum did significantly influence the degree of lipid accumulation, with more observed in HS treated cultures (Figure 1B, *p* =< 0.0001 *n* = 6, 15 FOV/rep). All subsequent work was performed in 10% HS.

**Figure 2:**
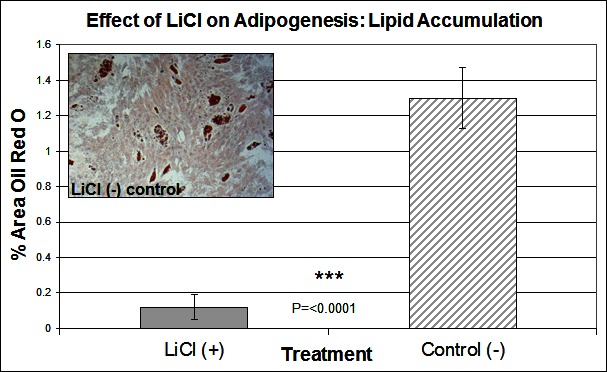
The effect of LiCl on adipogenesis. Lipid accumulation was measured by Oil Red O staining at Day 6 of differentiation, following treatment with LiCl (*n* = 6, 15 FOV/rep). Inset image representative of Oil Red O staining for control cells. Error bars represent SD.

**Figure 3:**
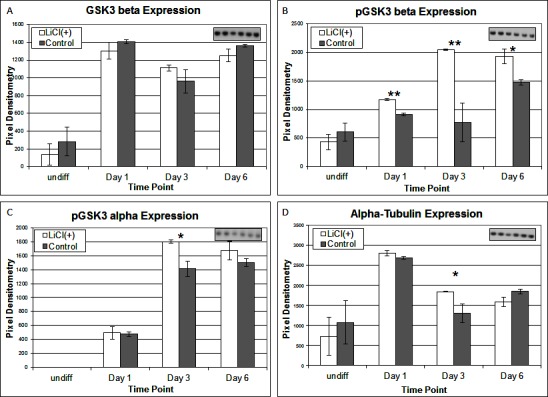
Expression of total GSK3-β **(A)**, pGSK3-β **(B)**, pGSK2-α **(C)** and α-Tubulin **(D)** proteins following LiCl treatment, at various time points of differentiation (*n* = 3). Inset images representative of western blot raw data. Error bars represent SD.

**Figure 4:**
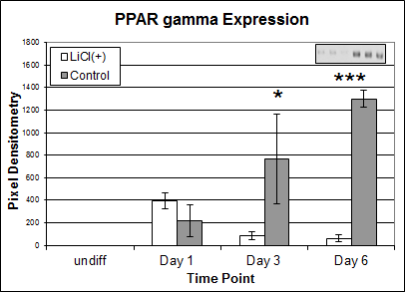
Effect of LiCl treatment on PPARγ expression. Expression of PPARγ protein following various time points of adipogenic differentiation (*n* = 3). Inset image representative of western blot raw data. Error bars represent SD.

To investigate the signalling pathways involved in the adipogenic cell fate of SM cells, we inhibited glycogen synthase kinase-3 (GSK-3) activity, effectively stimulating Wnt activation by addition of lithium chloride (LiCl). GSK-3 exists as two isoforms α and β both of which are expressed in skeletal muscle. These two isoforms show extensive homology and although they have similar functions they are not functionally redundant (20). It has been demonstrated that they have distinct roles in cardiogenesis with GSK-3α required for myocyte survival and GSK-3β modulating left right symmetry (21). To date GSK-3α is the much less studied of the two isoforms although some studies have shown it to be the more potent isoform. We have therefore also examined this isoform in the present study. Inhibition of GSK-3β by Lithium chloride has been shown to induce myotube hypertrophy in cells cultured in myogenic DM (10). In the present study in which cells were cultured in adipogenic DM lipid formation was virtually ablated in all treated cells, which was quantified by Oil Red O (ORO) staining (Figure 2B, *p* =< 0.0001 *n* = 6, 15 FOV/rep). Subsequently we examined the expression of both GSK3β and the inactivated phosphorylated form (pGSK3β), at the protein level, over several time points of differentiation: undifferentiated, Day 1, Day 3 and Day 6 in DM. Results for the level of total GSK3β showed no significant difference between treatment and control, at any time point (Figure 3A). In contrast, however, following initiation of differentiation, pGSK3β expression markedly increased in LiCl treated cells for all time points (Figure 3B, Day 1 *p* =< 0.001, Day 3 *p* =< 0.01, Day 6 *p* =< 0.05, *n* = 3), supporting the ORO results. Expression of the phosphorylated alpha form of GSK3 (pGSK3α) increased over time for both treatments but was only significantly higher in LiCl treated cells at Day 3 (Figure 3C, *p* =< 0.05, *n* = 3). Loading control, α-Tubulin expression was similar for samples at most time points (Figure 3D). Of note, we observe variable expression between treatments of many loading control proteins during early time points of myogenic and adipogenic differentiation including GAPDH, β-tubulin and β-actin). Following LiCl treatment, the key adipogenic regulatory transcription factor Peroxisome Proliferator Activator Protein gamma (PPARγ) was significantly down regulated from Day 3 of differentiation onwards, compared to the control (Figure 4, Day 3 *p* = 0.05, Day 6 *p* =< 0.001, *n* = 3). The fact that PPARγ was significantly down-regulated from day 3 and that ORO almost completely ablated by day 6 suggests that it is GSK3β and not GSK3α that plays the major role in this process. The down regulation of canonical wnt signalling with age and in certain disease processes suggests that this pathway may play a major role in the accumulation of adipose and its replacement of skeletal muscle tissue, in these conditions, in both large animals and humans.

## Conclusions

A number of studies have demonstrated that in both muscle cell lines (such as the murine C2C12 cells) and in primary stem cells derived from skeletal muscle that there is a limited potential for adipogenic trans-differentiation *in vitro*. We have previously shown that cells derived from different porcine skeletal muscles differ in their adipogenic potential and that furthermore this potential may be affected by the oxygen environment in which the cells are cultured. In the present study we have further optimised the conditions to promote adipogenic differentiation in porcine skeletal muscle derived stem cells. This adipogenic differentiation was almost completely prevented by the administration of lithium chloride (LiCl) which is a widely characterised inhibitor of GSK-3. This compound is in effect a mimic of Wnt pathway activation and the present study supports *in vivo* evidence that the Wnt pathway is inhibitory to adipose tissue formation.

## Author Contributions

Both Authors contributed to the design of the study, the interpretation of the data and the writing of the paper.

## Conflict of Interest Statement

The authors declare that the research was conducted in the absence of any commercial or financial relationships that could be construed as a potential conflict of interest.
